# Plant-Based Diet Quality, Healthy Lifestyle, and Dementia Risk in Older Adults With Cardiometabolic Diseases

**DOI:** 10.1016/j.jacadv.2025.102229

**Published:** 2025-10-08

**Authors:** Michelle M. Dunk, Abigail Dove, Jiao Wang, Sakura Sakakibara, Adrián Carballo-Casla, Weili Xu

**Affiliations:** a Aging Research Center, Department of Neurobiology, Care Sciences and Society, Karolinska Institutet, Stockholm, Sweden; b Division of Neurogeriatrics, Department of Neurobiology, Care Sciences and Society, Karolinska Institutet, Stockholm, Sweden; c National Clinical Research Center for Geriatrics, West China Hospital, Sichuan University, Chengdu, China; d CIBER of Epidemiology and Public Health (CIBERESP), Madrid, Spain

**Keywords:** heart disease, lifestyle, nutrition, stroke, type 2 diabetes, UK biobank

## Abstract

**Background:**

Cardiometabolic diseases (CMDs; heart disease, stroke, and type 2 diabetes) are associated with dementia risk, but whether a healthy plant-based diet and other lifestyle behaviors can mitigate this risk is unclear.

**Objectives:**

The purpose of this study was to investigate joint associations of CMDs, plant-based diet quality, and other lifestyle behaviors with dementia risk.

**Methods:**

Dementia-free UK Biobank participants aged ≥55 years with two or more 24-hour dietary recalls were included (N = 71,648). The plant-based diet index (PDI), healthful PDI (hPDI), and unhealthful PDI (uPDI) were calculated according to consumption of 17 food groups. Prevalent CMDs and incident dementia were determined from medical records. A healthy lifestyle beyond diet was defined as never smoking, nonheavy alcohol drinking, and high physical activity. Dementia risk was estimated using Cox regression.

**Results:**

A total of 9,656 (13.5%) participants had at least 1 CMD at baseline. After a median of 12.5 years, 825 (1.2%) participants developed dementia. Baseline CMDs were associated with significantly increased risk of dementia (HR: 1.90 [95% CI: 1.53-2.35]). Dementia risk associated with CMDs was reduced among those with high hPDI (HR: 0.39 [95% CI: 0.20-0.74]) and increased for those with high uPDI (HR: 3.24 [95% CI: 1.64-6.40]). The risk of dementia among participants with CMDs and low hPDI was attenuated in the presence of other healthy lifestyle behaviors (HR: 1.47 [95% CI: 0.34-6.36]).

**Conclusions:**

Dementia incidence among adults with CMDs differed substantially according to plant-based diet quality. Additional healthy lifestyle behaviors were associated with significant mitigation of dementia risk only in the presence of low hPDI.

The global prevalence of dementia is projected to reach over 150 million by 2050, almost triple the amount in 2019.^1^ Cardiometabolic diseases (CMDs) including heart disease, stroke, and type 2 diabetes are established risk factors for dementia.^2^^,^^3^ CMDs are widely prevalent and remain leading causes of death worldwide,^4^^,^^5^ posing a substantial barrier to efforts aimed at dementia prevention.^2^ Identification and dissemination of feasible intervention strategies for those already afflicted with CMDs is imperative to achieve a meaningful reduction in dementia rates.

A growing body of literature suggests the importance of a healthy diet for supporting both cardiometabolic and brain health. Mediterranean, Dietary Approaches to Stop Hypertension (DASH), and Mediterranean-DASH Intervention for Neurodegenerative Delay (MIND) diets have previously been associated with lower risk of developing both CMDs and dementia.^6^^,^^7^ These dietary patterns are higher in minimally processed (ie, *whole*) plant foods (fruits, vegetables, legumes, whole grains, nuts, and seeds) than the ubiquitous Western diet, which is high in animal-based and heavily processed foods.^8^ Yet intervention studies involving these diets have sometimes led to disappointing results,^9^^,^^10^ which could be due to moderate inclusion of some animal-based and heavily processed foods that have been linked to dementia risk.^11^, ^12^, ^13^

There is increasing evidence that a whole food, plant-based (WFPB) diet, which minimizes both animal-based and processed foods,^14^ may lead to considerable cardiometabolic and cognitive benefits. For instance, randomized trials have reported greater improvements in cardiometabolic risk factors in those consuming a predominantly WFPB diet compared to both the Mediterranean diet and a healthy omnivorous diet.^15^^,^^16^ Moreover, a WFPB diet has been shown to stop progression and cause regression of atherosclerosis and cardiovascular disease^17^^,^^18^ and lead to successful remission of type 2 diabetes.^19^ However, investigations of a WFPB diet in the context of dementia are scarce. Two recent prospective studies have reported lower dementia risk in relation to WFPB diet adherence,^20^ especially among men and carriers of the apolipoprotein E ε4 (*APOE*4) allele.^21^ Furthermore, a recent randomized controlled trial of Alzheimer’s disease patients reported improvements in cognition, function, and biomarkers in those consuming a WFPB diet in combination with other healthy lifestyle behaviors.^22^

Together, these findings pose an exciting prospect of dementia risk reduction through a WFPB diet, possibly due in part to the promotion of cardiometabolic health. Yet no study to date has examined the association of a WFPB diet with dementia risk in those with CMDs, a critically high-risk group. Whether adhering to a WFPB diet in combination with other healthy lifestyle behaviors might further lower dementia risk is also unclear. We therefore investigated the interplay between CMDs and plant-based diet quality in relation to dementia risk among older adults from the UK Biobank, and whether these associations are reinforced by additional lifestyle behaviors.

## Methods

### Study population

The UK Biobank is an ongoing large-scale, longitudinal study of over 500,000 middle-aged and older adults from the United Kingdom. Participants aged 40 to 70 years were recruited between 2006 and 2010 across the UK, and comprehensive baseline assessment visits took place across 22 assessment centers in England, Scotland, and Wales.^23^ The National Research Ethics Service of the National Health Service approved the UK Biobank’s data-collection procedures (Ref 21/NW/0157) in accordance with the Declaration of Helsinki, and all participants provided written informed consent.

For the current study, we excluded participants with prevalent dementia, type 1 diabetes, or missing information on CMD status at baseline (Supplemental Figure 1). We further excluded those missing dietary data, reporting extreme calorie intake (<600 or >3,500 kcal/day for women, and <800 or >4,200 kcal/day for men), or with only one dietary assessment, as recommended for diet-disease investigations using UK Biobank’s dietary data.^24^ Finally, we excluded those <55 years of age at baseline given that dementia onset is less common in younger individuals. The final study sample consisted of 71,648 participants.

### Dietary assessment

Participants reported their consumption of 206 different foods and 32 types of beverages over the past 24 hours using the Oxford WebQ, administered via touchscreen.^25^ Included participants completed at least 2 and up to 5 separate assessments between baseline (2009-2010) and 2012 (online cycle 1, February 2011 to April 2011; online cycle 2, June 2011 to September 2011; online cycle 3, October 2011 to December 2011; online cycle 4, April 2012 to June 2012). Validation of the Oxford WebQ has been published previously.^26^

Plant-based dietary adherence was assessed using 3 plant-based diet indices (PDIs) previously developed by Satija et al^27^—the PDI (reflecting overall intake of plant- vs animal-based foods), healthful PDI (hPDI) (indicating WFPB dietary adherence), and unhealthful PDI (uPDI) (assessing intake of more heavily processed plant foods)—according to existing evidence on associations of food groups with type 2 diabetes, cardiovascular disease, cancer, and related risk factors. Scores on each of these indices are calculated according to consumption of 18 food groups, including healthy plant foods (fruit, vegetables, whole grains, legumes, nuts, vegetable oils, and tea/coffee), less healthy plant foods (fruit juices, sugar-sweetened beverages, refined grains, potatoes, and sweets/desserts), and animal foods (animal fats, dairy, eggs, fish/seafood, meat [red meat and poultry], and miscellaneous animal-based foods). Information about total intake of vegetable oils was not available in this study, so scores were calculated from the remaining 17 food groups.

Participants' mean daily consumption of each food group was computed according to average intake across all available dietary assessments. Each food group was first divided into quartiles of consumption, excluding those with no consumption, and participants were assigned a corresponding score of 2 (lowest consumption), 3, 4, or 5 (highest consumption). Those with zero consumption were assigned a score of 1. Scores for all 3 PDIs were calculated by summing the scores of all 17 food groups, with reverse scores assigned to animal-based foods for all indices, less healthy plant-based foods for the hPDI, and healthy plant-based foods for the uPDI (Supplemental Table 1). In line with a previous UK Biobank study of dementia risk based on these indices, as well as other examinations of the PDIs, participants were grouped into quintiles of PDI, hPDI, and uPDI scores.^20^^,^^28^^,^^29^ These groups were then simplified to low (quintile 1), moderate (quintiles 2-4), and high (quintile 5) adherence to reduce the number of groups in analysis and streamline interpretation while still retaining the full range of dietary intake.

### Evaluation of CMD status at baseline

Diagnoses of CMDs at study entry, including heart disease (myocardial infarction, coronary artery disease, heart failure, atrial fibrillation, and angina), stroke, and type 2 diabetes, were obtained at baseline assessment visits. Heart disease and stroke were determined according to self-reported medical history and medical records. Participants were classified as having type 2 diabetes if they met any of the following criteria: medical record or self-reported history of type 2 diabetes, use of glucose-lowering medication, hemoglobin A1c ≥6.5%, or fasting plasma glucose ≥126 mg/dL. Recorded diagnoses were classified according to the International Classification of Diseases-9 and -10 codes (Supplemental Table 2).

### Ascertainment of dementia incidence

Information on dementia diagnosis was retrieved through self-reported medical history and linkage to hospital inpatient records and death registries (https://biobank.ndph.ox.ac.uk/ukb/ukb/docs/alg_outcome_dementia.pdf). A validation study of the accuracy of the UK Biobank’s dementia case ascertainment using this method reported a positive predictive value of 82.5%.^30^ Follow-up time was calculated from baseline to the date of dementia diagnosis, death, or the last available follow-up through January 20, 2022.

### Lifestyle behaviors

Information on participants' engagement in 3 additional modifiable lifestyle behaviors—physical activity, smoking, and alcohol intake—was collected at baseline. The International Physical Activity Questionnaire was used to assess physical activity according to metabolic equivalent of task (MET) minutes per week, and participants were categorized as low (<600 MET-min/week), moderate (600 to <3,000 MET-min/week), or high (≥3,000 MET-min/week).^31^ Smoking status was defined as never, former, or current smoking. Intake of alcoholic beverages (beer/cider, spirits, red wine, white wine, fortified wine, and other) was converted into total UK units per day (1 UK unit = 8 g ethanol).^32^ A healthy lifestyle was defined as high physical activity, never smoking, and avoidance of heavy alcohol drinking(<2 UK units of alcohol per day).^33^

### Covariate assessment

Age, sex, race (White vs non-White [Asian, Black, mixed, or other]), and education (college/university degree vs not) were collected at baseline. Socioeconomic status was quantified using the Townsend Deprivation Index according to neighborhood levels of unemployment, household overcrowding, car nonownership, and home nonownership, where higher scores indicate greater socioeconomic deprivation. Body mass index (BMI) (kg/m^2^) was calculated based on height and weight measurements. Hypertension was defined as self-reported hypertension, the use of antihypertensive medication, or baseline measurement of systolic blood pressure ≥140 mm Hg or diastolic blood pressure ≥90 mm Hg.

*APOE*4 carrier status was determined from genotyping using the Applied Biosystems UK BiLEVE Axiom array by Affymetrix and Applied Biosystems UK Biobank Axiom Array.^34^ Individuals were categorized as *APOE*4 carriers or noncarriers according to the presence of at least one ε4 allele at single-nucleotide polymorphisms rs7412 and rs429358. Full details of UK Biobank’s genotyping procedures are documented elsewhere.^35^

### Statistical analysis

Baseline characteristics according to hPDI groups were evaluated using analysis of variance (ANOVA) for continuous variables and chi-square tests for categorical variables. Associations between CMDs (combined CMD status, followed by heart disease, stroke, and type 2 diabetes individually) and dementia incidence were examined using Cox proportional hazards regression. Relationships of PDI, hPDI, and uPDI scores with dementia incidence were assessed using Cox regression. All models were adjusted for age, sex, race, education, socioeconomic status, BMI, smoking status, physical activity, alcohol intake, hypertension, and *APOE*4 status. Analysis of PDIs with dementia risk was also adjusted for CMD status and energy intake. Interactions of the PDIs with age (<65 vs ≥65 years), sex, BMI (<25 vs ≥25 kg/m^2^), CMD status, and *APOE*4 status were also examined, with PDI groups treated as an ordinal variable. Missing values for covariates were treated using missing indicator categories. Tests of Schoenfeld residuals were performed to evaluate proportional hazards. Race violated the proportional hazards assumption, so a time-dependent coefficient was used. A time-dependent coefficient was similarly used for stroke in the analysis of individual CMDs due to proportional hazards violation.

Dementia risk according to joint exposure to CMDs and each PDI was performed using Cox regression. A 6-category indicator variable was created based on CMD status and PDI groups, with the healthiest group as reference for each: 1) CMD-free + high PDI; 2) CMD-free + high hPDI; and 3) CMD-free + low uPDI. These models were repeated using CMDs + low diet groups as reference to obtain risk differences among those with CMDs. Sensitivity analyses were performed: 1) after excluding dementia cases in the first 5 years (n = 50) to account for potential reverse causality and missed cases of dementia during the beginning of follow-up; 2) in age-stratified groups of those aged <65 (n = 53,109) and ≥65 years (n = 18,539) to account for the increased dementia risk with age; and 3) across shorter time intervals (≤8, ≤10, and ≤12 years) to assess for regression dilution demonstrated in previous studies.^36^, ^37^, ^38^

We performed supplementary mediation analysis of key cardiometabolic risk factors (systolic and diastolic blood pressure, hemoglobin A1c, and BMI) in the association between each PDI and dementia for the full sample and CMD subset. Mediators were regressed on each continuous diet score using linear regression, followed by logistic regression of dementia on each mediator and diet score. β estimates and 95% confidence intervals of the total, direct, and indirect effects and proportion mediated were estimated using *mediate* in R Studio with bootstrapping (1,000 simulations). We additionally examined individual associations of the 17 food groups with dementia. Dementia risk according to quintiles of each food group was estimated using Cox regression first in the full sample, followed by CMD-stratified models. In the CMD subgroup, quintiles 1 and 2 of tea/coffee were combined due to an uneven distribution of scores. These models were also adjusted for hPDI scores calculated from the remaining 16 food groups.

We then explored the joint association of CMD status, hPDI, and a healthy lifestyle beyond diet with dementia risk. To maximize power, imputed data for missing lifestyle variables were used from multiple imputation by chained equations. A 12-category indicator variable of CMD status, hPDI, and lifestyle was created, with CMD-free + high hPDI + healthy lifestyle treated as the reference group. Comparison of dementia risk based on lifestyle within each CMD/diet group (CMD-free + moderate hPDI, CMD-free + low hPDI, CMDs + high hPDI, CMDs + moderate hPDI, CMDs + low hPDI) was then performed using unhealthy lifestyle as reference. To verify these results, we performed this analysis again using nonimputed data, excluding those missing data on at least one lifestyle variable (N = 5,738).

Analyses were performed using R Studio version 2023.06.1 + 524, 2009 to 2023, Posit Software, PBC, with two-tailed tests of statistical significance reported at *P* < 0.05.

## Results

### Participant characteristics

Characteristics of the study sample (N = 71,648) at baseline according to hPDI are presented in Table 1. The mean (SD) age at baseline was 61.6 (3.9) years. A total of 9,656 (13.5%) participants had at least one CMD at baseline (5,243 [7.3%] heart disease, 953 [1.3%] stroke, 4,696 [6.6%] type 2 diabetes). Participants were followed up for up to 15.1 years, from 2006 through 2022. After a median (SD) of 12.5 (1.5) years, 825 (1.2%) individuals developed dementia; 224 (27.2%) of whom had CMDs at baseline. Those with higher hPDI scores were more likely to be female, non-White, college-educated, nonsmokers, physically active, consume less alcohol, and have a lower BMI. Individuals with higher hPDI had lower prevalence of hypertension and CMDs and were more likely to have *APOE*4, lower energy intake, higher PDI, and lower uPDI scores. Scores on all dietary indices were normally distributed (Supplemental Figure 2). PDI, hPDI, and uPDI scores ranged from 32 to 77, 30 to 81, and 27 to 76, respectively. There were modest correlations between participants' first and last dietary scores (Pearson correlation coefficients for PDI, hPDI, and uPDI, respectively, were 0.37, 0.40, and 0.43, *P* < 0.001). Correlations between scores calculated from each assessment and averaged intake across assessments are detailed in Supplemental Table 3. Daily consumption of major food groups according to hPDI is displayed in Supplemental Table 4.

**Table 1:** Baseline Characteristics According to the hPDI

	hPDI			
	Pooled (n = 71,648)	Low (n = 14,500)	Moderate (n = 44,271)	High (n = 12,877)
Age (y)	61.6 ± 3.9	61.5 ± 3.9	61.7 ± 3.9	61.5 ± 3.9
Sex				
Male	33,713 (47.1)	8,752 (60.4)	20,487 (46.3)	4,474 (34.7)
Female	37,935 (52.9)	5,748 (39.6)	23,784 (53.7)	8,403 (65.3)
Race				
White	66,714 (93.1)	13,623 (94.0)	41,350 (93.4)	11,741 (91.2)
Non-white	4,696 (6.6)	831 (5.7)	2,785 (6.3)	1,080 (8.4)
College education	31,255 (43.6)	5,565 (38.4)	19,436 (43.9)	6,254 (48.6)
TDI	−1.9 ± 2.7	−1.8 ± 2 0.7	−1.9 ± 2.7	−1.8 ± 2.8
BMI (kg/m^2^)	26.9 ± 4.4	28.0 ± 4.7	26.8 ± 4.3	25.7 ± 4.1
Smoking				
Never	38,335 (53.5)	7,270 (50.1)	23,858 (53.9)	7,207 (56.0)
Former	29,053 (40.5)	5,998 (41.4)	17,917 (40.5)	5,138 (39.9)
Current	4,112 (5.7)	1,202 (8.3)	2,404 (5.4)	506 (3.9)
Physical activity				
Low	10,987 (15.3)	2,768 (19.1)	6,752 (15.3)	1,467 (11.4)
Moderate	26,705 (37.3)	5,426 (37.4)	16,524 (37.3)	4,755 (36.9)
High	22,897 (32.0)	4,016 (27.7)	14,145 (32.0)	4,736 (36.8)
Alcohol intake (U/day)	2.3 ± 2.3	2.6 ± 2.6	2.3 ± 2.3	1.9 ± 1.9
Hypertension	22,439 (31.3)	5,247 (36.2)	13,737 (31.0)	3,455 (26.8)
*APOE*4 carrier	16,372 (22.9)	3,137 (21.6)	10,079 (22.8)	3,156 (24.5)
CMDs	9,656 (13.5)	2,280 (15.7)	5,852 (13.2)	1,524 (11.8)
Heart disease	5,243 (7.3)	1,230 (8.5)	3,188 (7.2)	825 (6.4)
Stroke	953 (1.3)	254 (1.8)	569 (1.3)	130 (1.0)
Type 2 diabetes	4,696 (6.6)	1,153 (8.0)	2,810 (6.3)	733 (5.7)
PDI score	54.0 ± 5.4	51.3 ± 5.0	54.1 ± 5.5	57.1 ± 4.9
hPDI score	54.7 ± 6.3	46.1 ± 2.8	54.9 ± 3.0	64.0 ± 3.0
uPDI score	53.0 ± 6.0	56.7 ± 5.6	53.0 ± 5.5	48.4 ± 5.0
Total energy intake (kcal/day)	2,027 ± 447	2,220 ± 452	2,009 ± 432	1,869 ± 414

Values are mean ± SD or n (%). Heart disease includes myocardial infarction, atrial fibrillation, heart failure, coronary artery disease, and angina. Missing data: race = 238, education = 247, TDI = 67, BMI = 171, smoking = 148, physical activity = 11,059, alcohol intake = 6,631, hypertension = 69, *APOE*4 status = 12,179.

BMI = body mass index; CMD = cardiometabolic disease; hPDI = healthful plant-based diet index; PDI = plant-based diet index; TDI = Townsend Deprivation Index; uPDI = unhealthful plant-based diet index.

Characteristics between study participants, the entire UK Biobank, and excluded participants are presented in Supplemental Table 5. Compared to excluded participants, our sample has a lower incidence rate of dementia (1.2% vs 1.6%), and participants are more likely to be older, White race, to have less socioeconomic deprivation, and have a college education. Our sample is healthier than excluded participants in certain respects (lower BMI, lower prevalence of type 2 diabetes and stroke, fewer current smokers), while less healthy in others (higher prevalence of hypertension and heart disease).

### Individual associations between CMDs, PDIs, and dementia incidence

Associations of CMDs and the 3 PDIs with dementia risk are presented in Table 2. The presence of any CMD at baseline was associated with significantly higher incidence of dementia over follow-up (HR: 1.90 [95% CI: 1.53-2.35]). Heart disease (HR: 1.87 [95% CI: 1.47-2.38]), stroke (HR: 1.28 [95% CI: 1.03-1.59]), and type 2 diabetes (HR: 1.49 [95% CI: 1.11-2.01]) were each individually associated with increased dementia risk.

**Table 2:** Individual Associations of CMDs and the Plant-Based Diet Indices With Dementia Risk

Exposure	N	HR (95% CI)	*P* Value
CMD status			
Any CMD	9,656	1.90 (1.53-2.35)	<0.001
Heart disease	5,243	1.87 (1.47-2.38)	<0.001
Stroke	953	1.28 (1.03-1.59)	0.03
Type 2 diabetes	4,696	1.49 (1.11-2.01)	0.008
Plant-based diet quality			
PDI			
Low	14,110	Reference	
Moderate	42,988	0.80 (0.64-1.01)	0.06
High	14,550	0.95 (0.72-1.25)	0.71
hPDI			
Low	14,500	Reference	
Moderate	44,271	0.88 (0.71-1.11)	0.28
High	12,877	0.69 (0.51-0.95)	0.02
uPDI			
Low	13,004	Reference	
Moderate	45,823	1.56 (1.18-2.05)	0.002
High	12,821	2.11 (1.53-2.90)	<0.001

Models were adjusted for age, sex, race, education, socioeconomic status, BMI, smoking status, physical activity, alcohol intake, hypertension, and *APOE*4 status. Analyses of individual CMDs were mutually adjusted for the other CMDs. Analyses of the PDIs were also adjusted for energy intake and CMD status.

BMI = body mass index; CMD = cardiometabolic disease; hPDI = healthful plant-based diet index; HR = hazard ratio; PDI = plant-based diet index; uPDI = unhealthful plant-based diet index.

Dementia risk did not differ significantly by PDI (moderate: 0.80 [95% CI: 0.64-1.01]; high: 0.95 [95% CI: 0.72-1.25]). However, significant differences in dementia incidence were observed according to plant-based diet quality. Those with high hPDI had significantly lower risk of dementia (0.69 [95% CI: 0.51-0.95]) than those with low hPDI. Conversely, moderate (1.56 [95% CI: 1.18-2.05]) and high (2.11 [95% CI: 1.53-2.90]) uPDI were both associated with increased dementia risk.

There was a significant interaction between CMD status and hPDI, whereby dementia risk was lower in relation to higher hPDI among those with CMDs (0.72 [95% CI: 0.51-0.99], *P* for interaction = 0.04) than among CMD-free individuals (Supplemental Table 6). There were no interactions between CMD status and PDI or uPDI (*P* for interaction ≥0.20). No interactions were detected between the PDIs and age, sex, BMI, or *APOE*4 status (*P* for interaction ≥0.14).

### Joint association of CMD status and PDIs with dementia incidence

**Figure 1:**
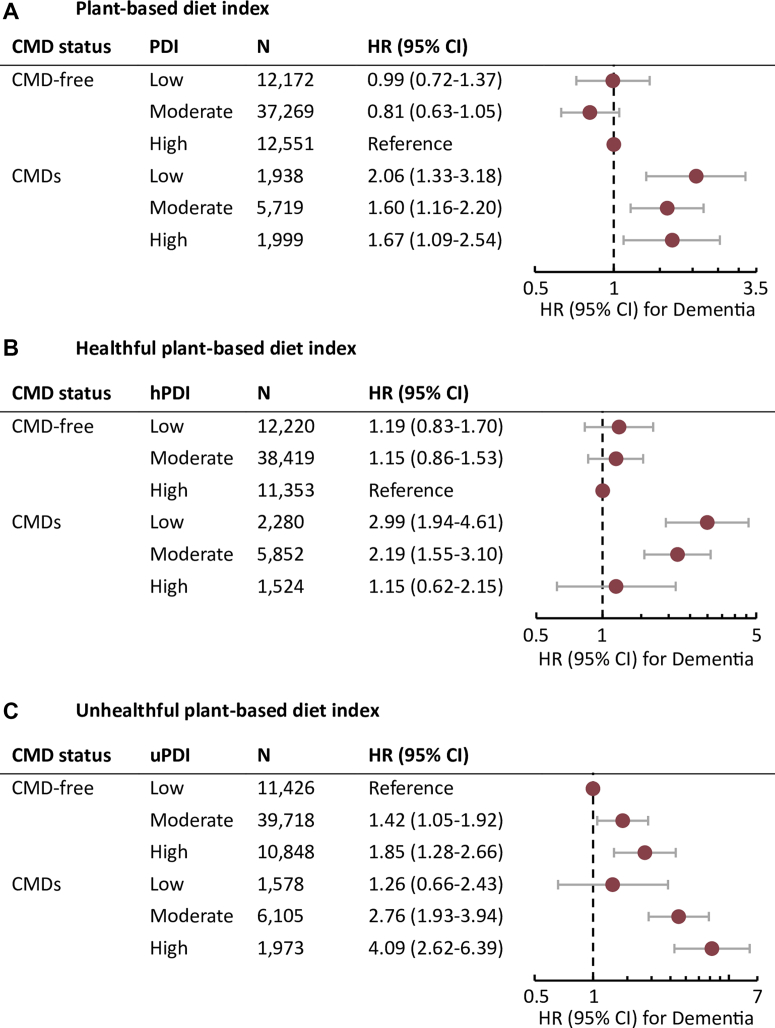
**Joint Associations of CMD Status and Plant-Based Diet Indices With Dementia Risk** Models were adjusted for age, sex, race, education, energy intake, socioeconomic status, BMI, smoking status, physical activity, alcohol intake, hypertension, and *APOE*4 status. BMI = body mass index; CMD = cardiometabolic disease; hPDI = healthful plant-based diet index; PDI = plant-based diet index; uPDI = unhealthful plant-based diet index.

Dementia risk based on CMD status and each PDI from joint exposure analysis is displayed in Figure 1. Compared to CMD-free individuals with high PDI, the hazard ratios for dementia among CMD participants with low or high PDI were 2.06 (95% CI: 1.33-3.18) and 1.67 (95% CI: 1.09-2.54), respectively. Dementia risk did not differ significantly for those with CMDs + high PDI (0.81 [95% CI: 0.48-1.36]) compared to those with CMDs + low PDI.

Compared to CMD-free + high hPDI, the hazard ratios for dementia among those with CMDs and low or high hPDI were 2.99 (95% CI: 1.94-4.61) and 1.15 (95% CI: 0.62-2.15), respectively. Those with CMDs and high hPDI had 61% lower dementia risk (0.39 [95% CI: 0.20-0.74]) than those with CMDs + low hPDI.

Compared to CMD-free + low uPDI, the hazard ratios for dementia among those with CMDs and low or high uPDI were 1.26 (95% CI: 0.66-2.43) and 4.09 (95% CI: 2.62-6.39), respectively. Among those with CMDs, high uPDI was associated with 3.24-fold higher dementia risk than low uPDI (3.24 [95% CI: 1.64-6.40]).

#### Sensitivity and supplementary analyses

Examinations of dementia risk according to individual CMDs and the 3 PDIs revealed similar trends, whereby dementia risk tended to decrease in relation to higher PDI, higher hPDI, and lower uPDI for those with heart disease, stroke, or type 2 diabetes (Supplemental Table 7). Joint associations of overall CMD status and PDIs with dementia risk remained very similar after excluding participants who developed dementia in the first 5 years of follow-up (Supplemental Table 8) and were somewhat stronger among those aged <65 compared to ≥65 years at baseline (Supplemental Table 9 and Supplemental Figure 3). Time interval-stratified analysis revealed a stronger association for hPDI at ≤8 years of follow-up (CMDs + high compared to low hPDI = 0.12 [95% CI: 0.01-0.91]), and for uPDI at ≤10 years (CMDs + high compared to low uPDI = 4.14 [95% CI: 1.55-11.08]) when compared to the full follow-up of 15.1 years (Supplemental Table 10 and Supplemental Figure 4). Mediation analysis indicated that hemoglobin A1c partially mediated the association between hPDI and dementia by 2.3% (direct effect: β: −0.001 [95% CI: −0.002 to −0.00003], *P* = 0.02; indirect effect: −0.00001 [95% CI: −0.00003 to −0.000001], *P* = 0.03; total effect: −0.001 [95% CI: −0.002 to −0.00004], *P* = 0.01) in the full sample, although not in the CMD subset (Supplemental Tables 11 and 12).

Several food groups were independently associated with dementia risk, with some variations by CMD status (Supplemental Figure 5, Supplemental Table 13). In the entire sample, high consumption of whole grains, vegetables, and tea/coffee was associated with reduced dementia risk, while intake of sugar-sweetened beverages and sweets/desserts was associated with increased dementia risk. In the CMD subset, the beneficial associations between vegetables and tea/coffee with reduced dementia risk remained, as did the harmful association between sugar-sweetened beverages and increased dementia risk. High intake of nuts was also linked to lower dementia risk in those with CMDs, while fruit juice was associated with higher dementia risk. Eggs were marginally associated with lower dementia risk in this group.

### Joint association of CMD status, hPDI, and a healthy lifestyle with dementia incidence

**Figure 2:**
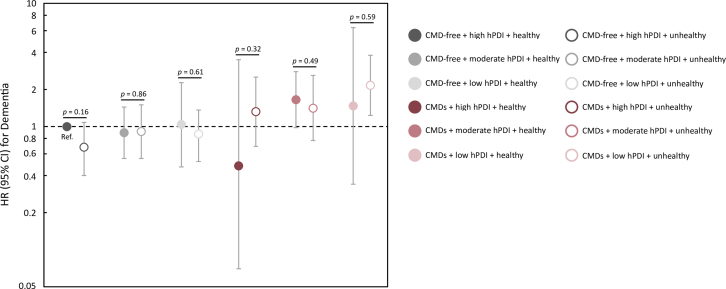
**Dementia Risk According to CMD Status, hPDI, and Healthy Lifestyle** A healthy lifestyle includes high physical activity + never smoking + low-to-moderate alcohol consumption. Models were adjusted for age, sex, race, education, energy intake, socioeconomic status, BMI, hypertension, and *APOE*4 status. Abbreviations: BMI = body mass index; CMD = cardiometabolic disease; hPDI = healthful plant-based diet index; Ref. = reference group.

Dementia risk according to CMD status and hPDI in the presence of other healthy lifestyle behaviors is shown in Figure 2 and Supplemental Table 14. The hazard ratio of dementia among those with CMDs + high hPDI was lower when combined with a healthy (0.48 [95% CI: 0.07-3.49]) as opposed to unhealthy (1.32 [95% CI: 0.69-2.52]) lifestyle. Dementia risk was highest among people with CMDs + low hPDI + unhealthy lifestyle (2.16 [95% CI: 1.23-3.79]), and this risk was attenuated in the presence of other healthy lifestyle behaviors (1.47 [95% CI: 0.34-6.36]). For those with CMDs, differences in dementia risk by lifestyle behaviors within hPDI groups did not reach significance (*P* ≥ 0.32). This could relate to limited power from small and uneven group sizes, so these results should be interpreted with caution. Results remained similar when excluding those missing information on lifestyle variables (Supplemental Table 15).

## Discussion

In this large prospective study of UK adults, higher adherence to a WFPB diet (reflected by high hPDI) was associated with 61% lower dementia risk among those with CMDs (Central Illustration). In contrast, people with CMDs consuming an unhealthy plant-based diet had 3.24-fold increased risk of dementia. The beneficial association between additional healthy lifestyle behaviors and lower CMD-associated dementia risk was most prominent among those not adhering to a WFPB diet. These findings expand on the growing literature pointing to the importance of consuming a diet rich in whole plant foods and low in processed and animal-based foods for reducing dementia risk. To our knowledge, our study is among the first to investigate dementia risk in relation to plant-based diet quality among those with CMDs, an especially vulnerable group.

**Central Illustration:**
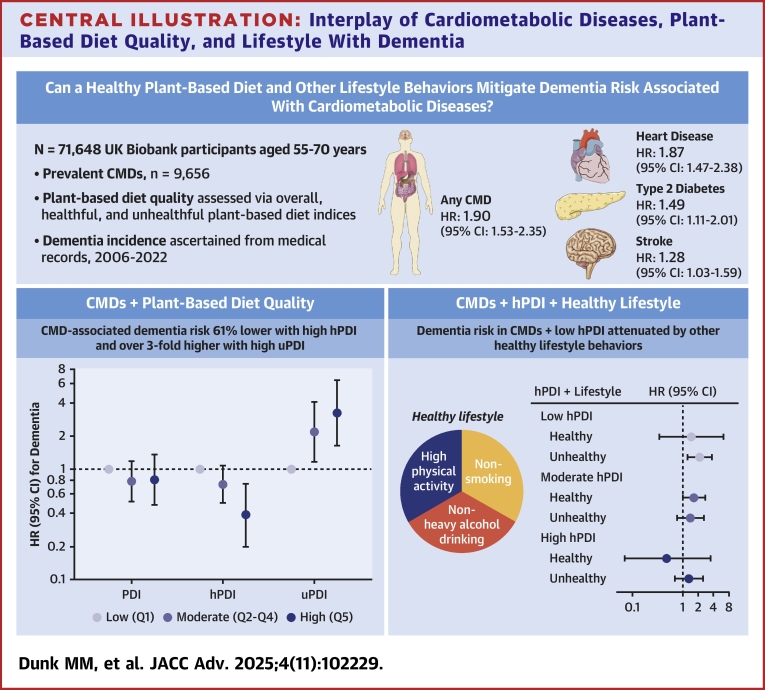
**Interplay of Cardiometabolic Diseases, Plant-Based Diet Quality, and Lifestyle With Dementia** Prevalent CMDs including heart disease, type 2 diabetes, and stroke were associated with increased dementia risk among older UK Biobank participants. The risk of dementia among those with CMDs was reduced with high hPDI and increased with high uPDI. Dementia risk for those with CMDs and low hPDI was attenuated in the presence of other healthy lifestyle behaviors (high physical activity, nonsmoking, and nonheavy alcohol drinking). Image attributions: Male body with organs. Reactome, release 88, https://reactome.org/content/detail/R-ICO-013956. Heart-front, pancreas, and brain-2 by Servier https://smart.servier.com/is licensed under CC-BY 3.0 Unported https://creativecommons.org/licenses/by/3.0/. CMD = cardiometabolic disease; hPDI = healthful plant-based diet index; hPDI = healthful plant-based diet index; Q = quintile; uPDI = unhealthful plant-based diet index.

### Plant-based diet quality and dementia risk in CMDs

Two prospective studies examining associations between the PDIs and dementia risk have reported results somewhat similar to ours. Higher hPDI and lower uPDI scores, although not overall PDI scores, were associated with reduced incidence of dementia in 180,532 middle-aged and older UK Biobank participants.^20^ Unlike our study, this prior UK Biobank investigation did not consider the interplay between CMDs and plant-based diet quality in the context of dementia, and also did not examine other healthy lifestyle behaviors.^20^ In a sample of 9,543 adults aged ≥55 years from the Rotterdam Study, higher hPDI scores were associated with lower dementia risk only among men and *APOE*4 carriers.^21^ The consistent lack of association between overall PDI scores and dementia risk in our study and the 2 prior reports^20^^,^^21^ is revealing, implying that general plant-based diet consumption may not be enough to mitigate dementia risk. Rather, the *quality* of plant-based diet—one that consists primarily of whole plant foods and minimizes less healthy, more processed plant foods—seems essential. This is further supported by reports of elevated dementia risk in relation to higher consumption of ultraprocessed foods (eg, sugary beverages and packaged, ready-to-eat foods)^13^ and some animal-based foods, especially processed and red meat.^11^^,^^12^

It is also worth noting that associations of hPDI and uPDI with dementia risk were strongest among adults <65 years of age and at shorter follow-up times. While the age difference could partly reflect greater statistical power in the younger group (n = 53,109 vs n = 18,539), the observed risk variation by age and follow-up time is consistent with prior studies.^36^, ^37^, ^38^ Attenuation of effect estimates with age and longer follow-up may be explained by regression dilution bias^36^^,^^38^ due to dietary changes and accumulating risk factors with age. Previous studies of plant-based diet quality and dementia risk involved long follow-up periods (median of 10 years^20^ and mean of 14.5 years^21^), which may have similarly led to underestimation of dementia risk. This underscores the importance of incorporating repeated dietary assessments in future diet-dementia investigations to obtain more accurate risk estimates.^36^^,^^38^

Higher hPDI and lower uPDI scores have previously been associated with significantly lower risk of CMDs, although their association with CMD-related dementia risk has not been reported.^27^^,^^39^^,^^40^ Our group previously found that an anti-inflammatory diet, measured by the nutrient-based Dietary Inflammatory Index, was associated with lower dementia risk among older adults with CMDs.^41^ A WFPB diet is theoretically one of the most anti-inflammatory diets^14^^,^^42^^,^^43^ and can be more readily translated into accessible dietary recommendations than the Dietary Inflammatory Index due its emphasis on whole foods rather than micronutrients and macronutrients. In the current study, dementia risk among older adults with CMDs was substantially attenuated for those with high hPDI or low uPDI, and this risk increased considerably with lower hPDI and higher uPDI scores. These trends underscore the possibility for dementia prevention through a WFPB diet among those with poor cardiometabolic health. Interestingly, among CMD-free individuals, higher uPDI but not lower hPDI scores were associated with increased dementia risk. The stronger associations of both hPDI and uPDI scores with dementia among those with CMDs could allude to cardiometabolic health as a major intermediary factor linking plant-based diet quality with dementia development. This possibility is further supported by the partial mediation of hPDI and dementia risk by hemoglobin A1c, although future investigations involving repeated measurements are needed for verification.

A number of mechanisms may underpin the association between plant-based diet quality and CMD-associated dementia risk. Consumption of saturated fat (present in animal products, tropical oils, and many ultraprocessed foods) and cholesterol (found exclusively in animal products) has been shown to increase the burden of Alzheimer disease pathology in animal models of the disease.^44^^,^^45^ Many animal-based and processed foods promote oxidative stress and systemic inflammation—key drivers of CMD and Alzheimer disease pathogenesis—due to harmful compounds such as advanced glycation end-products^46^ and initiation of trimethylamine N-oxide production in the gut.^47^^,^^48^ While fish is anti-inflammatory and tends to be associated with lower dementia risk,^49^ it is also a primary source of exposure to toxic industrial pollutants^50^^,^^51^ implicated in the pathology of cardiovascular disease and dementia, including heavy metals, microplastics and nanoplastics, and perfluoroalkyl and polyfluoroalkyl substances.^52^, ^53^, ^54^, ^55^, ^56^ Aside from limiting exposure to these harmful substances, a WFPB diet is rich in dietary fiber, antioxidants, and anti-inflammatory nutrients that support a myriad of cardiometabolic functions closely tied to cognitive health, including endothelial function, glycemic control, insulin sensitivity, weight management, blood lipids, and blood pressure.^57^, ^58^, ^59^

### Role of other lifestyle behaviors

Additional lifestyle behaviors aside from diet may also be important for dementia risk modification.^2^ Several prior investigations have detected lower dementia risk among people with cardiovascular disease, type 2 diabetes, or ≥2 CMDs adhering to a combination of healthy lifestyle factors.^60^, ^61^, ^62^ These include former/never smoking, moderate to vigorous physical activity, moderate alcohol intake, adequate sleep, minimal sedentary time, frequent social contact, and BMI <30 kg/m^2^ in combination with a healthy diet (high intake of fruits, vegetables, high-fiber bread, whole grains, and fish; low intake of refined grains and meat).^60^, ^61^, ^62^ We found that healthy lifestyle behaviors (high physical activity, nonsmoking, and avoidance of heavy alcohol drinking) were associated with lower dementia risk among those with CMDs and a diet high in animal-based and processed foods. Interestingly, for those with CMDs consuming a WFPB diet, additional healthy lifestyle behaviors were only related to a nonsignificant reduction of dementia risk. This could suggest that a WFPB diet has a greater ability to modify CMD-associated dementia risk than other lifestyle factors. However, for those with CMDs who are not adhering to this diet, engaging in other healthy lifestyle behaviors may then play a larger role in dementia risk modification.

### Strengths and limitations

Strengths of this study include the large prospective sample, consideration of diet-dementia associations in the context of CMDs, and exploration of dementia risk in the presence of diet and other modifiable lifestyle behaviors. Our examination of CMD-related dementia risk in relation to plant-based diet quality provides a unique addition to the dementia literature, which has historically focused on other dietary patterns. Nevertheless, several limitations should also be acknowledged. The primary reliance on medical records for diagnosis of CMDs and dementia introduces the possibility of underdiagnosis, particularly considering that individuals with milder cases may have been less likely to seek a diagnosis. In regards to dementia, our sensitivity analysis excluding dementia cases within the first 5 years alleviates some risk of underdiagnosis of early-stage dementia.

The use of self-reported dietary data comes with an inherent risk of recall bias. While information on long-term dietary intake was not available in this sample past 2012, we minimized this limitation by using averaged consumption data from 2 to 5 assessments per individual between 2009 and 2012. Subsequent changes in dietary habits have been reported in older age^63^ and preceding dementia diagnosis,^64^ which were not captured in this study. The food group classification of the PDIs also contains some limitations when distinguishing between animal- and plant-based foods. For instance, potatoes are often consumed with butter or other animal-derived foods, while sweets/desserts frequently contain milk, eggs, and other animal byproducts. The likelihood that high intake of potatoes and sweets/desserts also reflects animal product consumption should therefore be acknowledged when interpreting results.

Finally, the UK Biobank is not representative of the broader UK population due to healthy volunteer selection bias,^65^ and our sample also has a lower incidence of dementia than the entire UK Biobank. A majority (74%) of participants were <65 years of age at study enrollment, which could have contributed to the relatively low dementia incidence. Given that dementia risk increases with age, future investigations involving older participants are needed. These characteristics limit the generalizability of our findings and could have led to underestimation of associations.

## Conclusions

This large prospective study reveals significantly reduced dementia risk in older adults with CMDs consuming a predominantly WFPB diet. These novel findings signify the importance of encouraging those in poor cardiometabolic health to incorporate more whole plant foods in place of processed and animal-based foods for reducing the risk of developing dementia. Engaging in additional healthy lifestyle behaviors, such as physical activity and avoidance of smoking and heavy alcohol intake, may further decrease dementia risk in those with CMDs, especially for those consuming an unhealthy diet.

Perspectives **COMPETENCY IN MEDICAL KNOWLEDGE AND PATIENT CARE:** Plant-based diet quality may play a significant role in dementia risk modification, especially among older adults with CMDs. Encouraging this high-risk patient population to replace unhealthy plant foods with whole grains, vegetables, fruits, legumes, nuts, tea, and coffee and to engage in additional healthy lifestyle behaviors could support dementia prevention.

**TRANSLATIONAL OUTLOOK:** Clinical trials are needed to confirm causality between plant-based diet quality, other lifestyle behaviors, and dementia risk. The extent to which plant-based diet quality may modify dementia risk through cardiometabolic risk factors also needs to be elucidated in future research.

## Funding support and author disclosures

Dr Xu has received grants from the 10.13039/501100004359Swedish Research Council (2021-01647), the Swedish Council for Health, Working Life and Welfare (2021-01826), Karolinska Institutet Research Foundation (2022), and 10.13039/100019796Demensfonden (2023-2024). Dr Dunk has received support from Ragnhild and Einar Lundström’s Memorial Foundation via Lindhés Advokatbyrå AB (LA2024-0059, LA2025-0015) and 10.13039/100019796Demensfonden (2024). All other authors have reported that they have no relationships relevant to the contents of this paper to disclose.
