# Supplementary material for: Plant-Based Diet Quality, Healthy Lifestyle, and Dementia Risk in Older Adults With Cardiometabolic Diseases
Source: JACC Adv. 2025 Oct 8;4(11):102229. doi: 10.1016/j.jacadv.2025.102229 (PMC12546824; doi:10.1016/j.jacadv.2025.102229)
Supplement: Supplemental_Appendix [file mmc1.pdf]

Supplemental Appendix

Supplemental Figure 1. Study flowchart.

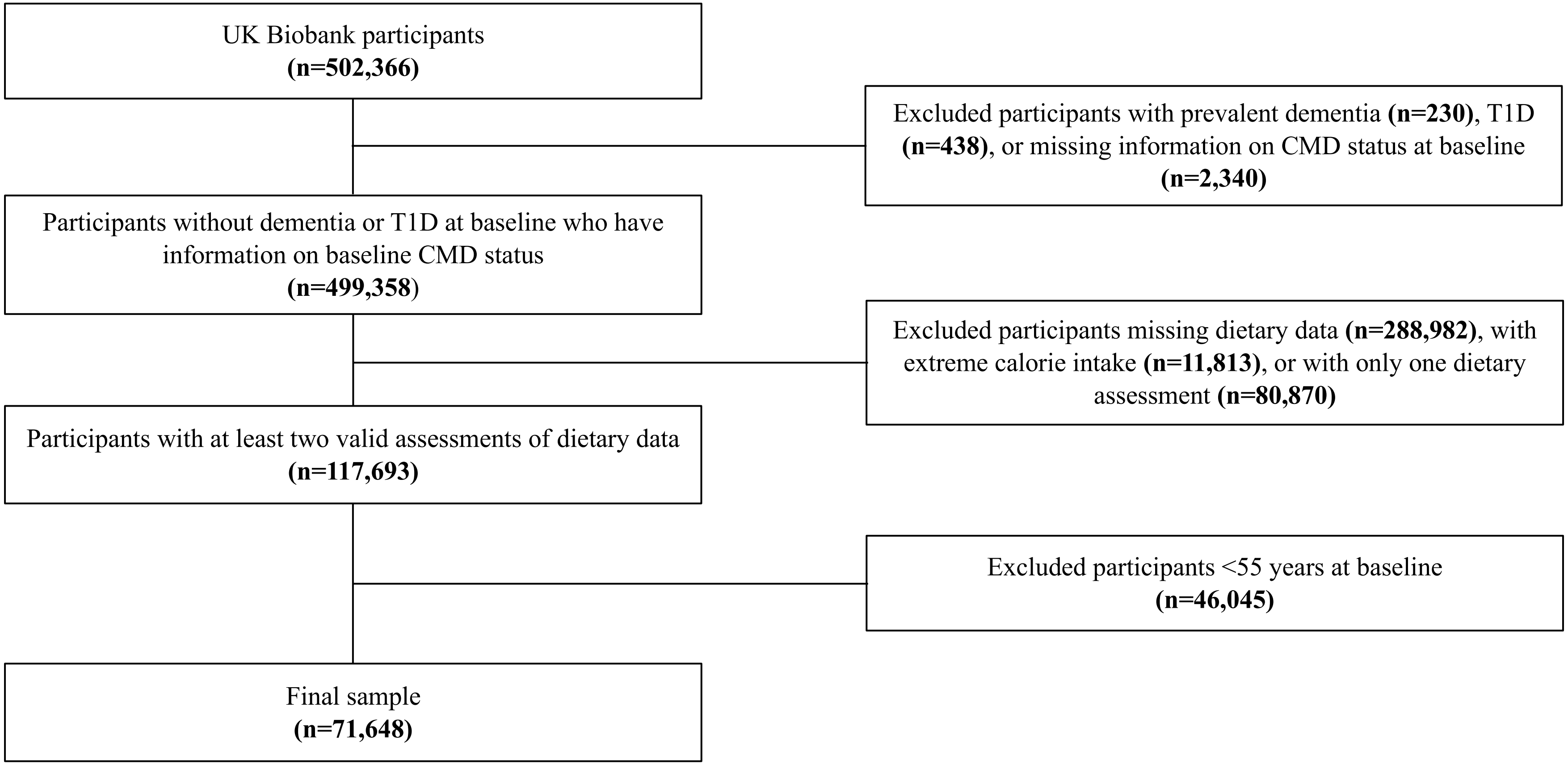

Abbreviations: CMD = cardiometabolic disease; T1D = type 1 diabetes.

Supplemental Table 1. Food items used to calculate the plant-based diet indices.

Food group	Food items	Scoring PDI	hPDI	uPDI
Healthy plant foods		Positive	Positive	Reverse
Fruits	Apples, bananas, berries, cherries, grapes, mango, melon, grapefruit, orange, satsuma, peaches/nectarines, pears, pineapple, plums, prunes, mixed fruit, stewed fruit, other fruit, dried fruit			
Vegetables	Beetroot, broccoli, butternut squash, cabbage/kale, carrots, cauliflower, celery, courgette, cucumber, garlic, leek, lettuce, mushroom, onion, parsnip, sweet peppers, spinach, sprouts, sweetcorn, sweet potato, fresh tomatoes, tinned tomatoes, turnip, watercress, mixed vegetables, vegetable pieces, coleslaw, side salad, other vegetables			
Whole grains	Non-white bread (sliced bread, baps, baguettes, bread rolls; types: wholemeal, seeded, mixed, other), porridge, muesli, oat crunch, bran cereal, cereal bars, crispbread, whole wheat cereal, other cereal, wholemeal pasta, brown rice, couscous, other grains			
Nuts	Salted peanuts, unsalted peanuts, salted nuts, unsalted nuts, seeds			
Legumes and vegetarian protein alternatives	Baked beans, broad beans, green beans, peas, other beans/lentils,, soy or other vegetable milk (including glasses/cartons of milk, milk used to make porridge, lattes, and cappuccinos, and milk added to coffee, tea, and cereal), vegetarian sausages/burgers, tofu, Quorn, other vegetarian alternatives (e.g. nut roast)			
Tea and coffee	Instant coffee, filtered coffee, cappuccino, espresso, latte, other coffee, standard tea, green tea, herbal tea, rooibos tea, other tea			
Less healthy plant foods		Positive	Reverse	Positive
Refined grains	White bread (sliced bread, baps, baguettes, bread rolls), naan bread, garlic bread, white pasta, white rice, plain cereal, sweetened cereal, pancakes, scotch pancakes, croissants, scones, cheesy biscuits, savory biscuits, savory snacks, snackpots			
Potatoes	Fried potatoes, boiled/baked potatoes, mashed potatoes, crisps			
Fruit juices	Orange juice, grapefruit juice, other pure fruit/vegetable juice, fruit smoothie			
Sugary drinks	Carbonated (fizzy) drinks, squash or cordial, low calorie/diet drinks			
Sweets and desserts	Double crust pie, single crust pie, crumble, Yorkshire pudding, Danish pastry, fruitcake, cake, doughnuts, sponge pudding, chocolate bars, white chocolate, milk chocolate, dark chocolate, chocolate raisins, chocolate sweets, diet sweets, chocolate covered biscuits, chocolate biscuits, sweet biscuits, sweets, other desserts, other sweets, soya desserts			
Animal foods		Reverse	Reverse	Reverse
Animal fat	Butter spreads on bread/crackers, dairy spreads on bread/crackers			
Dairy	Milk (cow's, goat's, or sheep's milk; including glasses/cartons of milk/flavored milk, milk used to make porridge, lattes, and cappuccinos, and milk added to coffee, tea, and cereal), dairy smoothie, yogurt, ice cream,			

	low fat hard cheese, hard cheese, soft cheese, blue cheese, low fat cheese spread, cheese spread, cottage cheese, feta cheese, mozzarella cheese, goat cheese, other cheese, cheesecake, milk pudding, other milk pudding
Eggs	Whole eggs, omelettes/scrambled eggs, egg sandwiches, scotch egg, other egg dishes
Fish and seafood	Tinned tuna, oily fish, breaded fish, battered fish, white fish, prawns, lobster/crab, shellfish, other fish
Meat	Beef, pork, lamb, poultry, crumbed/deep-fried poultry, sausage, bacon, ham, liver, other meat
Miscellaneous animal-based foods	Pizza, Indian snacks

Abbreviations: hPDI = healthful plant-based diet index; PDI = plant-based diet index; uPDI = unhealthful plant-based diet index.

Supplemental Table 2. UK Biobank field codes used to classify baseline CMD status.

Disease	Medical records (ICD-10 codes)	Self-reported medical history	Self-reported medications	Biochemical measures
Type 2 diabetes	130709 (E11)	2443	6153	30750 (HbA1c ≥6.5%)
	130711 (E12)	2976	6177	
	130713 (E13)			
	130715 (E14)			
Heart disease	131297 (I20)	3627		
	131299 (I21)	3894		
	131301 (I22)	6150		
	131303 (I23)			
	131305 (I24)			
	131307 (I25)			
	131351 (I48)			
	131353 (I49)			
	131355 (I50)			
Stroke	131361 (I60)	4056		
	131363 (I61)			
	131365 (I62)			
	131367 (I63)			
	131375 (I67)			
	131377 (I68)			

Supplemental Figure 2. Histograms of the plant-based diet indices.

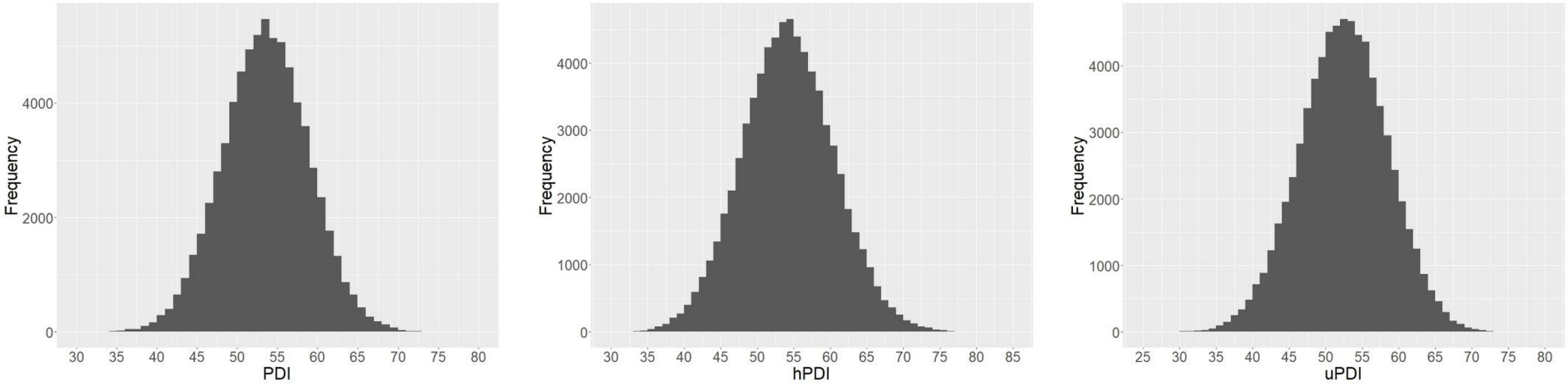

Abbreviations: hPDI = healthful plant-based diet index; PDI = plant-based diet index; uPDI = unhealthful plant-based diet index.

Supplemental Table 3. Pearson correlation coefficients of scores on the plant-based diet indices across assessments.

A. PDI

	Baseline	Cycle 1	Cycle 2	Cycle 3	Cycle 4	Average
**N**	19,919	48,161	42,849	52,760	49,725	71,648
**Mean ± SD**	43.86 ± 6.36	44.04 ± 6.24	44.42 ± 6.33	43.69 ± 6.18	44.15 ± 6.29	53.8 ± 5.19
**Baseline**	1	0.35	0.34	0.33	0.34	0.31
**Cycle 1**		1	0.39	0.39	0.37	0.31
**Cycle 2**			1	0.38	0.39	0.32
**Cycle 3**				1	0.39	0.30
**Cycle 4**					1	0.30
**Average**						1

B. hPDI

	Baseline	Cycle 1	Cycle 2	Cycle 3	Cycle 4	Average
**N**	19,919	48,161	42,849	52,760	49,725	71,648
**Mean ± SD**	47.73 ± 6.46	47.35 ± 6.55	47 53 ± 6.60	47.15 ± 6.52	47.40 ± 6.56	55.73 ± 6.11
**Baseline**	1	0.38	0.37	0.36	0.37	0.40
**Cycle 1**		1	0.41	0.42	0.40	0.41
**Cycle 2**			1	0.41	0.41	0.41
**Cycle 3**				1	0.41	0.41
**Cycle 4**					1	0.40
**Average**						1

C. uPDI

	Baseline	Cycle 1	Cycle 2	Cycle 3	Cycle 4	Average
**N**	19,919	48,161	42,849	52,760	49,725	71,648
**Mean ± SD**	45.11 ± 6.70	45.81 ± 6.67	45.91 ± 6.68	45.80 ± 6.69	46.06 ± 6.72	52.36 ± 5.84
**Baseline**	1	0.43	0.42	0.43	0.41	0.40
**Cycle 1**		1	0.45	0.46	0.44	0.41
**Cycle 2**			1	0.46	0.44	0.42
**Cycle 3**				1	0.45	0.41
**Cycle 4**					1	0.40
**Average**						1

Supplemental Table 4. Daily intake of food groups based on hPDI.

	Healthful Plant-Based Diet Index		
**Food groups (servings/day)**	**Low**	**Moderate**	**High**
Healthy plant foods			
Fruit	1.55 ± 1.22	2.36 ± 1.49	3.35 ± 1.68
Vegetables	1.76 ± 1.37	2.49 ± 1.67	3.54 ± 2.10
Whole grains	1.77 ± 1.26	2.41 ± 1.34	2.92 ± 1.40
Nuts	0.06 ± 0.20	0.14 ± 0.30	0.34 ± 0.47
Legumes	0.26 ± 0.33	0.40 ± 0.42	0.67 ± 0.57
Tea and coffee	4.05 ± 1.59	4.52 ± 1.58	4.95 ± 1.63
Less healthy plant foods			
Refined grains	1.76 ± 1.15	1.01 ± 0.91	0.57 ± 0.66
Potatoes	0.87 ± 0.51	0.70 ± 0.49	0.51 ± 0.45
Sugary drinks	0.71 ± 0.85	0.37 ± 0.64	0.16 ± 0.42
Fruit juice	0.57 ± 0.54	0.46 ± 0.51	0.32 ± 0.46
Sweets and desserts	1.93 ± 1.30	1.43 ± 1.14	0.93 ± 0.94
Animal foods			
Dairy	1.83 ± 0.84	1.79 ± 0.84	1.68 ± 0.91
Eggs	0.44 ± 0.46	0.28 ± 0.40	0.18 ± 0.35
Meat	1.45 ± 0.84	1.12 ± 0.75	0.77 ± 0.67
Fish and seafood	0.38 ± 0.40	0.34 ± 0.38	0.30 ± 0.39
Animal fat	1.10 ± 1.13	0.57 ± 0.90	0.25 ± 0.60
Miscellaneous animal foods	0.13 ± 0.35	0.06 ± 0.23	0.03 ± 0.15

Values are M ± SD.

Supplemental Table 5. Characteristics of the entire UK Biobank, excluded participants, and current study sample.

Characteristic	UK Biobank population	Excluded participants	Study sample
N	502,366	430,718	71,648
Age at baseline (y)	56.5 ± 8.1	55.7 ± 8.3	61.6 ± 3.9
Sex			
Male	229,066 (45.6)	195,353 (45.4)	33,713 (47.1)
Female	273,300 (54.4)	235,365 (54.6)	37,935 (52.9)
Race			
White	454,133 (90.4)	387,419 (89.9)	66,714 (93.1)
Non-white	45,457 (9.0)	40,761 (9.5)	4,696 (6.6)
College education	162,523 (32.4)	131,268 (30.5)	31,255 (43.6)
TDI	-1.3 ± 3.1	-1.2 ± 3.1	-1.9 ± 2.7
BMI (kg/m^2)	27.4 ± 4.8	27.5 ± 4.9	26.9 ± 4.4
Smoking			
Never	273,449 (54.4)	235,114 (54.6)	38,335 (53.5)
Former	173,008 (34.4)	143,955 (33.4)	29,053 (40.5)
Current	52,961 (10.5)	48,849 (11.3)	4,112 (5.7)
Physical activity			
Low	76,190 (15.2)	65,203 (15.1)	10,987 (15.3)
Moderate	163,987 (32.6)	137,282 (31.9)	26,705 (37.3)
High	162,096 (32.3)	139,199 (32.3)	22,897 (32.0)
Alcohol intake (units/day)	2.4 ± 2.6	2.4 ± 2.6	2.3 ± 2.3
Hypertension	143,663 (28.6)	121,224 (28.1)	22,439 (31.3)
Heart disease	34,192 (6.8)	28,949 (6.7)	5,243 (7.3)
Stroke	7,667 (1.5)	6,714 (1.6)	953 (1.3)
Type 2 diabetes	37,160 (7.4)	32,464 (7.5)	4,696 (6.6)
Dementia	7,896 (1.6)	7,071 (1.6)	835 (1.2)
*APOE*4 carrier	117,845 (23.5)	101,473 (23.6)	16,372 (22.9)

Values are M ± SD or N (%). Heart disease includes myocardial infarction, atrial fibrillation, heart failure, coronary artery disease, and angina.

Abbreviations: BMI = body mass index; CMDs = cardiometabolic diseases; TDI = Townsend Deprivation Index.

Supplemental Table 6. Interactions between the plant-based diet indices and age, sex, body mass index, CMD status, and *APOE*4 status in relation to dementia risk.

Plant-based diet quality	Interaction variable	HR (95% CI)	*p*-interaction
PDI	Age	1.04 (0.78-1.38)	0.80
	Sex	1.24 (0.93-1.65)	0.14
	BMI	0.89 (0.67-1.19)	0.45
	CMD status	1.01 (0.85-1.20)	0.47
	*APOE*4 status	0.95 (0.72-1.26)	0.73
hPDI	Age	0.80 (0.60-1.08)	0.14
	Sex	0.91 (0.68-1.23)	0.55
	BMI	0.88 (0.65-1.18)	0.39
	CMD status	0.72 (0.51-0.99)	0.04
	*APOE*4 status	0.96 (0.72-1.29)	0.80
uPDI	Age	1.13 (0.83-1.52)	0.44
	Sex	0.95 (0.70-1.29)	0.76
	BMI	1.12 (0.82-1.52)	0.48
	CMD status	1.24 (0.89-1.74)	0.20
	*APOE*4 status	1.06 (0.78-1.42)	0.72

Models were adjusted for race, education, energy intake, socioeconomic status, smoking status, physical activity, alcohol intake, and hypertension, and mutually adjusted for age, sex, body mass index, CMD status, and *APOE*4 status.

Abbreviations: BMI = body mass index; CI = confidence interval; CMD = cardiometabolic disease; hPDI = healthful plant-based diet index; HR = hazard ratio; PDI = plant-based diet index; uPDI = unhealthful plant-based diet index.

Supplemental Table 7. Joint association of individual CMDs and the plant-based diet indices with dementia risk.

CMDs/PDI	HR (95% CI)	*p*-value	CMDs/hPDI	HR (95% CI)	*p*-value	CMDs/uPDI	HR (95% CI)	*p*-value
Heart disease-free			Heart disease-free			Heart disease-free		
Low	1.04 (0.76-1.41)	0.82	Low	1.33 (0.95-1.87)	0.10	Low	Reference	
Moderate	0.83 (0.65-1.06)	0.13	Moderate	1.19 (0.90-1.58)	0.22	Moderate	1.55 (1.17-2.05)	0.002
High	Reference		High	Reference		High	2.12 (1.53-2.94)	<0.001
Heart disease			Heart disease			Heart disease		
Low	1.96 (1.16-3.29)	0.01	Low	2.69 (1.63-4.45)	<0.001	Low	1.72 (0.42-7.08)	0.45
Moderate	1.63 (1.14-2.32)	0.007	Moderate	2.39 (1.65-3.46)	<0.001	Moderate	3.28 (1.78-6.04)	<0.001
High	1.70 (1.06-2.71)	0.03	High	1.23 (0.61-2.49)	0.57	High	3.25 (1.17-8.99)	0.02
Stroke-free			Stroke-free			Stroke-free		
Low	1.03 (0.77-1.36)	0.85	Low	1.40 (1.01-1.92)	0.04	Low	Reference	
Moderate	0.85 (0.68-1.06)	0.15	Moderate	1.25 (0.96-1.62)	0.10	Moderate	1.49 (1.11-1.99)	0.008
High	Reference		High	Reference		High	1.85 (1.30-2.62)	<0.001
Stroke			Stroke			Stroke		
Low	3.23 (1.40-7.42)	0.006	Low	3.05 (1.30-7.13)	0.01	Low	1.22 (0.55-2.69)	0.62
Moderate	1.41 (0.71-2.80)	0.33	Moderate	2.69 (1.45-5.01)	0.002	Moderate	2.62 (1.78-3.85)	<0.001
High	1.59 (0.58-4.34)	0.36	High	0.65 (0.09-4.68)	0.67	High	4.80 (3.00-7.68)	<0.001
T2D-free			T2D-free			T2D-free		
Low	1.06 (0.79-1.43)	0.70	Low	1.41 (1.01-1.96)	0.04	Low	Reference	
Moderate	0.88 (0.70-1.11)	0.29	Moderate	1.28 (0.97-1.68)	0.08	Moderate	1.52 (1.14-2.03)	0.004
High	Reference		High	Reference		High	2.19 (1.57-3.05)	<0.001
T2D			T2D			T2D		
Low	1.86 (1.03-3.36)	0.04	Low	2.39 (1.33-4.30)	0.003	Low	1.41 (0.60-3.28)	0.43
Moderate	1.16 (0.75-1.80)	0.50	Moderate	1.88 (1.22-2.90)	0.004	Moderate	2.69 (1.77-4.10)	<0.001
High	1.81 (1.02-3.19)	0.04	High	1.36 (0.58-3.15)	0.48	High	2.09 (0.98-4.45)	0.06

Models were adjusted for age, sex, race, education, energy intake, socioeconomic status, body mass index, smoking status, physical activity, alcohol intake, hypertension, *APOE*4 status, and the other CMDs not being examined. Models were mutually adjusted for heart disease, stroke, and T2D.

Abbreviations: CI = confidence interval; CMD = cardiometabolic disease; hPDI = healthful plant-based diet index; HR = hazard ratio; PDI = plant-based diet index; T2D = type 2 diabetes; uPDI = unhealthful plant-based diet index.

Supplemental Table 8. Joint association of CMD status and the plant-based diet indices with dementia risk after excluding dementia cases occurring within the first 5 years of follow-up.

**CMDs/PDI**	**HR (95% CI)**	***p*-value**	**CMDs/hPDI**	**HR (95% CI)**	***p*-value**	**CMDs/uPDI**	**HR (95% CI)**	***p*-value**
CMD-free			CMD-free			CMD-free		
Low	0.93 (0.67-1.29)	0.67	Low	1.11 (0.78-1.60)	0.56	Low	Reference	
Moderate	0.75 (0.58-0.97)	0.03	Moderate	1.07 (0.80-1.44)	0.63	Moderate	1.40 (1.03-1.91)	0.03
High	Reference		High	Reference		High	1.82 (1.25-2.64)	0.002
CMDs			CMDs			CMDs		
Low	1.76 (1.11-2.80)	0.02	Low	2.82 (1.81-4.38)	<0.001	Low	1.20 (0.61-2.38)	0.60
Moderate	1.49 (1.07-2.07)	0.02	Moderate	2.01 (1.41-2.86)	0.001	Moderate	2.66 (1.84-3.84)	<0.001
High	1.67 (1.09-2.54)	0.02	High	1.16 (0.62-2.17)	0.65	High	4.16 (2.64-6.56)	<0.001

Models were adjusted for age, sex, race, education, energy intake, socioeconomic status, body mass index, smoking status, physical activity, alcohol intake, hypertension, and *APOE*4 status.

Abbreviations: CI = confidence interval; CMD = cardiometabolic disease; hPDI = healthful plant-based diet index; HR = hazard ratio; PDI = plant-based diet index; uPDI = unhealthful plant-based diet index.

Supplemental Table 9. Joint association of CMD status and the plant-based diet indices with dementia risk stratified by age at baseline.

	PDI		hPDI		uPDI	
**CMDs/PDI**	**HR (95% CI)**	***p*-value**	**HR (95% CI)**	***p*-value**	**HR (95% CI)**	***p*-value**
<65 years						
CMD-free						
Low	0.95 (0.57-1.57)	0.83	0.87 (0.49-1.56)	0.64	Reference	
Moderate	0.86 (0.57-1.29)	0.46	1.20 (0.78-1.85)	0.41	1.27 (0.80-2.01)	0.31
High	Reference		Reference		1.42 (0.81-2.48)	0.22
CMDs						
Low	2.08 (1.02-4.24)	0.04	3.41 (1.76-6.62)	<0.001	1.21 (0.42-3.53)	0.73
Moderate	1.81 (1.07-3.03)	0.03	2.29 (1.32-3.96)	0.003	2.50 (1.43-4.40)	0.001
High	1.88 (0.92-3.84)	0.08	1.00 (0.35-2.88)	1.00	4.31 (2.19-8.50)	<0.001
≥65 years						
CMD-free						
Low	1.04 (0.68-1.58)	0.86	1.46 (0.92-2.31)	0.11	Reference	
Moderate	0.78 (0.56-1.08)	1.14	1.12 (0.76-1.66)	0.57	1.54 (1.03-2.33)	0.03
High	Reference		Reference		2.25 (1.39-3.64)	0.001
CMDs						
Low	2.05 (1.18-3.58)	0.01	2.81 (1.58-4.98)	<0.001	1.35 (0.59-3.11)	0.48
Moderate	1.50 (0.99-2.26)	0.05	2.20 (1.40-3.47)	0.001	2.98 (1.87-4.76)	<0.001
High	1.59 (0.94-2.68)	0.08	1.27 (0.58-2.78)	0.54	4.19 (2.31-7.58)	<0.001

Models were adjusted for age, sex, race, education, energy intake, socioeconomic status, body mass index, smoking status, physical activity, alcohol intake, hypertension, and *APOE*4 status.

Abbreviations: CI = confidence interval; CMD = cardiometabolic disease; hPDI = healthful plant-based diet index; HR = hazard ratio; PDI = plant-based diet index; uPDI = unhealthful plant-based diet index.

Supplemental Figure 3. Dementia risk by plant-based diet indices across age groups in those with CMDs.

A. Plant-based diet index

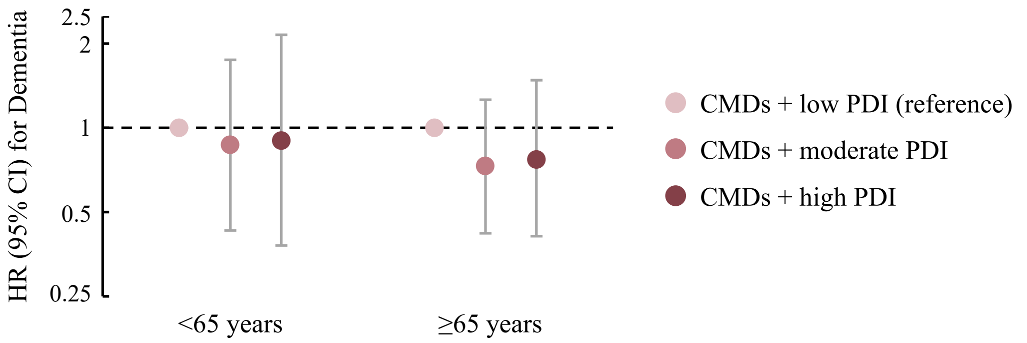

B. Healthful plant-based diet index

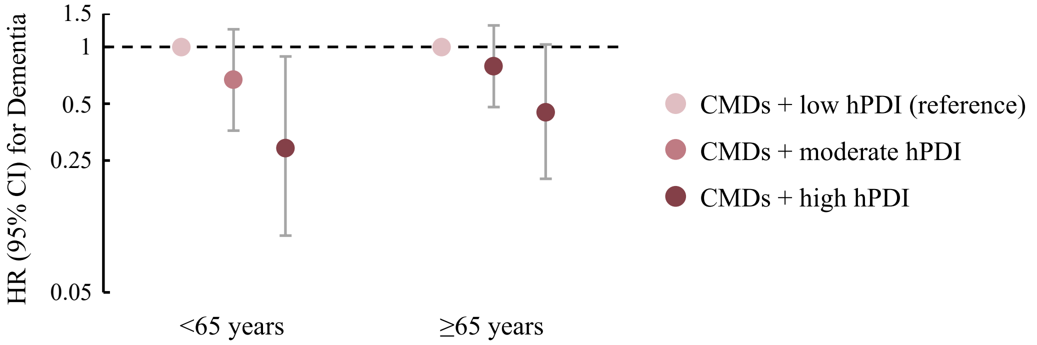

C. Unhealthful plant-based diet index

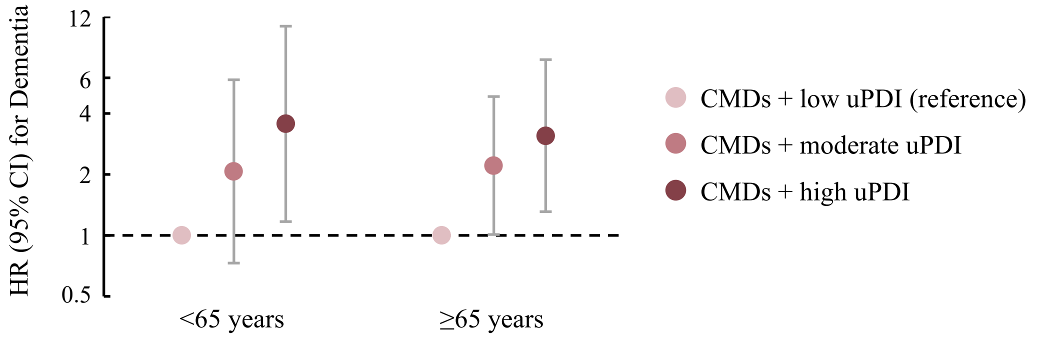

Hazard ratios and 95% confidence intervals are from fully adjusted joint exposure models, with A) CMDs + low PDI, B) CMDs + low hPDI, and C) CMDs + low uPDI as reference.

Abbreviations: CI = confidence interval; CMD = cardiometabolic disease; hPDI = healthful plant-based diet index; HR = hazard ratio; PDI = plant-based diet index; uPDI = unhealthful plant-based diet index.

Supplemental Table 10. Joint association of CMD status and the plant-based diet indices with dementia risk at shorter follow-up intervals.

	PDI		hPDI		uPDI	
**CMDs/PDI**	**HR (95% CI)**	***p*-value**	**HR (95% CI)**	***p*-value**	**HR (95% CI)**	***p*-value**
≤8 years						
CMD-free						
Low	1.13 (0.58-2.20)	0.72	1.67 (0.75-3.73)	0.21	Reference	
Moderate	0.82 (0.48-1.42)	0.48	1.61 (0.81-3.18)	0.17	1.45 (0.76-2.79)	0.26
High	Reference		Reference		1.80 (0.83-3.93)	0.14
CMDs						
Low	3.11 (1.39-6.96)	0.006	4.65 (1.84-11.73)	0.001	2.03 (0.64-6.46)	0.23
Moderate	1.84 (0.95-3.59)	0.07	3.94 (1.84-8.42)	<0.001	2.99 (1.41-6.34)	0.004
High	1.94 (0.84-4.48)	0.12	0.53 (0.07-4.21)	0.55	5.62 (2.35-13.45)	<0.001
≤10 years						
CMD-free						
Low	1.14 (0.71-1.81)	0.59	1.41 (0.82-2.42)	0.21	Reference	
Moderate	0.95 (0.65-1.38)	0.78	1.54 (0.99-2.40)	0.05	1.52 (0.98-2.36)	0.06
High	Reference		Reference		1.87 (1.11-3.17)	0.02
CMDs						
Low	2.27 (1.20-4.28)	0.01	3.88 (2.05-7.33)	<0.001	1.21 (0.46-3.19)	0.70
Moderate	1.94 (1.23-3.08)	0.005	3.25 (1.95-5.41)	<0.001	3.34 (2.02-5.54)	<0.001
High	2.60 (1.52-4.47)	0.001	1.67 (0.71-3.91)	0.24	5.01 (2.70-9.28)	<0.001
≤12 years						
CMD-free						
Low	0.94 (0.67-1.33)	0.73	1.24 (0.84-1.82)	0.28	Reference	
Moderate	0.76 (0.58-1.00)	0.047	1.19 (0.87-1.62)	0.29	1.63 (1.16-2.30)	0.005
High	Reference		Reference		2.11 (1.40-3.16)	<0.001
CMDs						
Low	2.00 (1.26-3.18)	0.003	2.99 (1.86-4.78)	<0.001	1.45 (0.72-2.91)	0.30
Moderate	1.40 (0.98-1.98)	0.06	2.18 (1.49-3.17)	<0.001	2.98 (1.99-4.45)	<0.001
High	1.60 (1.02-2.49)	0.04	1.09 (0.55-2.16)	0.81	4.40 (2.68-7.24)	<0.001

Models were adjusted for age, sex, race, education, energy intake, socioeconomic status, body mass index, smoking status, physical activity, alcohol intake, hypertension, and *APOE*4 status.

Abbreviations: CI = confidence interval; CMD = cardiometabolic disease; hPDI = healthful plant-based diet index; HR = hazard ratio; PDI = plant-based diet index; uPDI = unhealthful plant-based diet index.

Supplemental Figure 4. Dementia risk by plant-based diet indices across follow-up intervals in those with CMDs.

A. Plant-based diet index

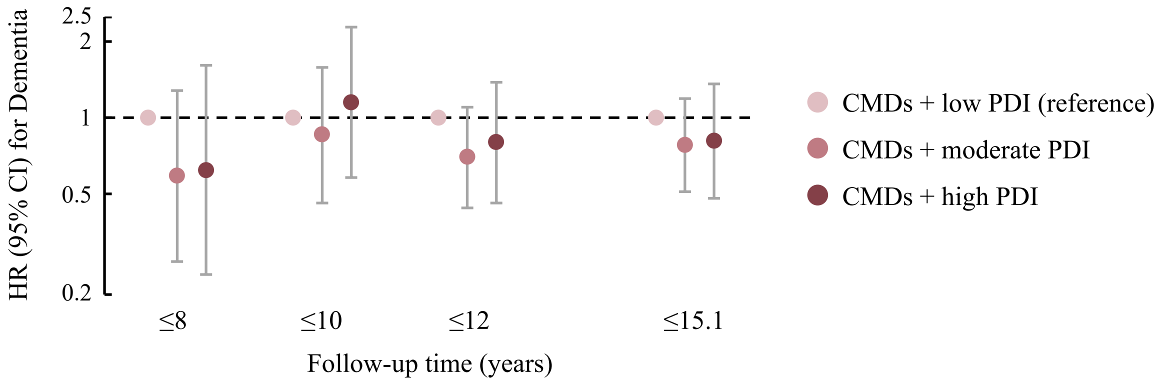

B. Healthful plant-based diet index

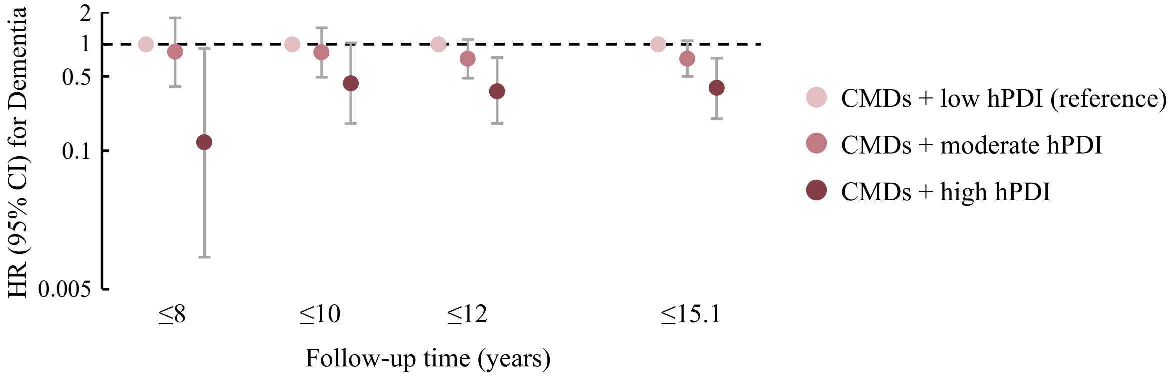

C. Unhealthful plant-based diet index

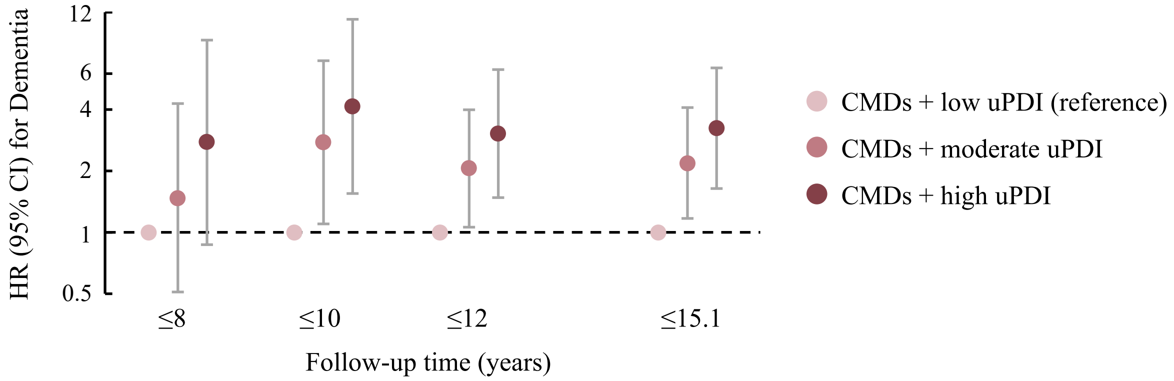

Hazard ratios and 95% confidence intervals are from fully adjusted joint exposure models, with A) CMDs + low PDI, B) CMDs + low hPDI, and C) CMDs + low uPDI as reference.

Abbreviations: CI = confidence interval; CMD = cardiometabolic disease; hPDI = healthful plant-based diet index; HR = hazard ratio; PDI = plant-based diet index; uPDI = unhealthful plant-based diet index.

Supplemental Table 11. Mediating role of cardiometabolic risk factors in the association between the plant-based diet indices and dementia risk.

Mediators	Total effect, β (95% CI)	Indirect effect, β (95% CI)	Direct effect, β (95% CI)	Proportion mediated (%)
PDI				
Systolic blood pressure	-0.00002 (-0.001, 0.0001)	0.000003 (-0.000001, 0.00001)	-0.00002 (-0.001, 0.0001)	NA
Diastolic blood pressure	-0.00002 (-0.001, 0.0001)	0.0000002 (-0.000002, 0.000003)	-0.00002 (-0.001, 0.0001)	NA
HbA1c	-0.00003 (-0.001, 0.0001)	-0.000001 (-0.000004, 0.000002)	-0.00003 (-0.001, 0.0001)	NA
Body mass index	-0.00002 (-0.001, 0.0001)	-0.000004 (-0.00003, 0.00001)	-0.00001 (-0.001, 0.0001)	NA
hPDI				
Systolic blood pressure	-0.001 (-0.002, -0.0001)**	-0.000004 (-0.00001, 0.000001)	-0.001 (-0.002, -0.0001)**	NA
Diastolic blood pressure	-0.001 (-0.002, -0.0001)*	0.00001 (-0.00001, 0.00003)	-0.001 (-0.002, -0.0001)**	NA
HbA1c	-0.001 (-0.002, -0.00004)*	-0.00001 (-0.00003, -0.000001)*	-0.001 (-0.002, -0.00003)*	2.30%
Body mass index	-0.0001 (-0.002, -0.0001)*	-0.00001 (-0.0001, 0.0001)	-0.001 (-0.002, -0.0001)*	NA
uPDI				
Systolic blood pressure	0.0001 (0.00003, 0.0001)***	0.0000001 (-0.0000001, 0.000001)	0.0001 (0.00003, 0.0001)***	NA
Diastolic blood pressure	0.0001 (0.00003, 0.0001)***	-0.0000002 (-0.000001, 0.0000001)	0.0001 (0.00003, 0.0001)***	NA
HbA1c	0.0001 (0.00004, 0.0001)***	0.0000001 (-0.0000002, 0.0000005)	0.0001 (0.00004, 0.0001)***	NA
Body mass index	0.0001 (0.00004, 0.0001)***	0.0000002 (-0.000001, 0.000001)	0.0001 (0.00004, 0.0001)***	NA

Models were adjusted for age, sex, race, education, energy intake, socioeconomic status, smoking status, physical activity, alcohol intake, and *APOE*4 status. *$p < 0.05$, **$p < 0.01$, ***$p < 0.001$.

Abbreviations: CI = confidence interval; HbA1c = hemoglobin A1c; hPDI = healthful plant-based diet index; NA = not applicable due to no significant mediation effect; PDI = plant-based diet index; uPDI = unhealthful plant-based diet index.

Supplemental Table 12. Mediating role of cardiometabolic risk factors in the association between the plant-based diet indices and dementia risk among participants with CMDs.

Mediators	Total effect, β (95% CI)	Indirect effect, β (95% CI)	Direct effect, β (95% CI)	Proportion mediated (%)
PDI				
Systolic blood pressure	-0.001 (-0.01, 0.0002)	0.00002 (-0.00001, 0.0001)	-0.001 (-0.01, 0.0002)	NA
Diastolic blood pressure	-0.001 (-0.01, 0.0002)	-0.000004 (-0.00004, 0.00002)	-0.001 (-0.01, 0.0002)	NA
HbA1c	-0.001 (-0.01, 0.0002)	0.00001 (-0.00002, 0.0001)	-0.001 (-0.01, 0.0002)	NA
Body mass index	-0.001 (-0.01, 0.0002)	-0.00001 (-0.0002, 0.0002)	-0.001 (-0.01, 0.0002)	NA
hPDI				
Systolic blood pressure	-0.01 (-0.02, -0.001)***	0.0001 (-0.00004, 0.0002)	-0.01 (-0.02, -0.001)***	NA
Diastolic blood pressure	-0.01 (-0.02, -0.001)**	-0.00003 (-0.0002, 0.0001)	-0.01 (-0.02, -0.001)**	NA
HbA1c	-0.01 (-0.01, -0.001)**	0.0001 (-0.00003, 0.0002)	-0.01 (-0.01, -0.001)**	NA
Body mass index	-0.01 (-0.02, -0.001)***	0.0001 (-0.001, 0.001)	-0.01 (-0.02, -0.001)***	NA
uPDI				
Systolic blood pressure	0.0001 (0.00002, 0.0001)***	-0.0000003 (-0.000002, 0.000001)	0.0001 (0.00002, 0.0002)***	NA
Diastolic blood pressure	0.0001 (0.00002, 0.0001)***	0.0000002 (-0.000001, 0.000002)	0.0001 (0.00002, 0.0001)***	NA
HbA1c	0.0001 (0.00002, 0.0002)**	0.000001 (-0.000001, 0.000005)	0.0001 (0.00002, 0.0002)**	NA
Body mass index	0.0001 (0.00001, 0.0001)***	0.0000001 (-0.000003, 0.000004)	0.0001 (0.00001, 0.0001)***	NA

Models were adjusted for age, sex, race, education, energy intake, socioeconomic status, smoking status, physical activity, alcohol intake, and *APOE*4 status. *$p < 0.05$, **$p < 0.01$, ***$p < 0.001$.

Abbreviations: CI = confidence interval; HbA1c = hemoglobin A1c; hPDI = healthful plant-based diet index; NA = not applicable due to no significant mediation effect; PDI = plant-based diet index; uPDI = unhealthful plant-based diet index.

Supplemental Figure 5. Associations of individual food groups with dementia risk.

Food group	HR* (95% CI)	*p*-trend
Fruit	0.88 (0.55-1.42)	0.83
Vegetables	0.56 (0.34-0.92)	0.01
Whole grains	0.58 (0.36-0.94)	<0.001
Nuts	0.90 (0.62-1.31)	0.82
Legumes	0.88 (0.67-1.14)	0.19
Tea and coffee	0.40 (0.18-0.91)	<0.001
Refined grains	0.99 (0.73-1.34)	0.89
Potatoes	0.87 (0.64-1.19)	0.52
Fruit juice	1.18 (0.86-1.61)	0.42
Sugary drinks	1.43 (1.07-1.91)	0.02
Sweets and desserts	1.57 (1.04-2.39)	0.03
Animal fat	1.00 (0.76-1.32)	0.24
Dairy	1.31 (0.42-4.15)	0.69
Eggs	0.84 (0.64-1.12)	0.15
Fish and seafood	0.92 (0.69-1.22)	0.84
Meat	0.79 (0.56-1.12)	0.24
Miscellaneous animal foods	1.11 (0.67-1.85)	0.45

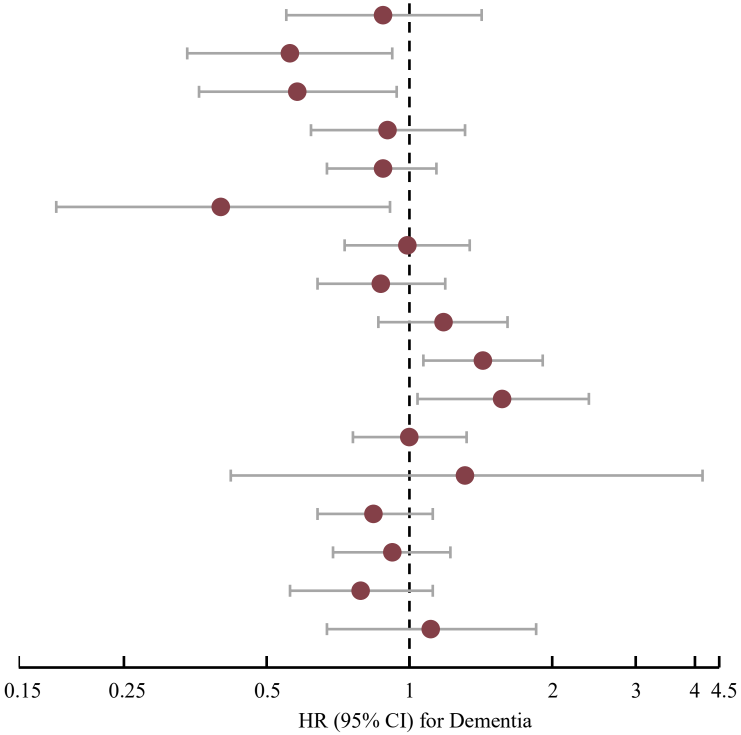

Models were adjusted for age, sex, race, education, energy intake, socioeconomic status, body mass index, smoking status, physical activity, alcohol intake, hypertension, CMD status, *APOE*4 status, and hPDI scores excluding the food group being examined.

*Hazard ratio of dementia for highest vs lowest consumption.

Abbreviations: CI = confidence interval; CMD = cardiometabolic disease; hPDI = healthful plant-based diet index; HR = hazard ratio.

Supplemental Table 13. Associations of individual food groups with dementia risk according to CMD status.

Food group	CMD-free, N = 61,992<br>HR* (95% CI)	*p*-trend	CMDs, N = 9,656<br>HR* (95% CI)	*p*-trend
Fruit	0.75 (0.43-1.32)	0.74	1.31 (0.54-3.21)	0.38
Vegetables	0.96 (0.44-2.11)	0.10	0.33 (0.16-0.68)	0.02
Whole grains	0.57 (0.33-1.01)	0.007	0.58 (0.21-1.56)	0.06
Nuts	1.11 (0.75-1.65)	0.64	0.24 (0.06-0.97)	0.20
Legumes	0.89 (0.65-1.22)	0.41	0.82 (0.51-1.33)	0.23
Tea and coffee	0.34 (0.15-0.78)	0.01	0.53 (0.32-0.88)	0.02
Refined grains	0.91 (0.64-1.29)	0.71	1.31 (0.70-2.45)	0.72
Potatoes	0.91 (0.63-1.32)	0.62	0.77 (0.44-1.37)	0.75
Fruit juice	0.97 (0.66-1.43)	0.82	1.87 (1.08-3.25)	0.049
Sugary drinks	1.18 (0.81-1.73)	0.26	1.89 (1.19-3.01)	0.02
Sweets and desserts	1.66 (1.00-2.75)	0.02	1.43 (0.68-3.00)	0.66
Animal fat	0.93 (0.67-1.29)	0.10	1.14 (0.68-1.92)	0.85
Dairy	1.41 (0.34-5.74)	0.37	1.38 (0.19-10.18)	0.42
Eggs	0.93 (0.67-1.29)	0.63	0.64 (0.37-1.12)	0.05
Fish and seafood	0.92 (0.66-1.27)	0.77	0.93 (0.54-1.61)	0.94
Meat	0.85 (0.56-1.28)	0.11	0.63 (0.33-1.21)	0.74
Miscellaneous animal foods	1.00 (0.53-1.89)	0.52	1.41 (0.61-3.28)	0.65

Models were adjusted for age, sex, race, education, energy intake, socioeconomic status, body mass index, smoking status, physical activity, alcohol intake, hypertension, *APOE*4 status, and hPDI scores excluding the food group being examined.

*Hazard ratio of dementia for highest vs lowest consumption.

Abbreviations: CI = confidence interval; CMD = cardiometabolic disease; hPDI = healthful plant-based diet index; HR = hazard ratio.

Supplemental Table 14. Joint association of CMD status, hPDI, and healthy lifestyle with dementia risk using imputed data for missing lifestyle variables.

CMD status	hPDI	Lifestyle score	N	Dementia HR (95% CI)	*p*-value
CMD-free	Low	Healthy	1,231	1.04 (0.47-2.27)	0.93
		Unhealthy	10,989	0.87 (0.52-1.46)	0.59
	Moderate	Healthy	27,480	0.89 (0.55-1.44)	0.62
		Unhealthy	10,939	0.91 (0.55-1.50)	0.70
	High	Healthy	1,981	Reference	
		Unhealthy	9,372	0.68 (0.40-1.16)	0.16
CMDs	Low	Healthy	153	1.47 (0.34-6.36)	0.61
		Unhealthy	2,127	2.16 (1.23-3.79)	0.008
	Moderate	Healthy	4,201	1.65 (0.98-2.79)	0.06
		Unhealthy	1,651	1.41 (0.77-2.60)	0.27
	High	Healthy	207	0.48 (0.07-3.49)	0.47
		Unhealthy	1,317	1.32 (0.69-2.52)	0.40

A healthy lifestyle includes high physical activity + never smoking + low-to-moderate alcohol consumption. Models were adjusted for age, sex, race, education, energy intake, socioeconomic status, body mass index, hypertension, and *APOE*4 status.

Abbreviations: CI = confidence interval; CMD = cardiometabolic disease; hPDI = healthful plant-based diet index; HR = hazard ratio.

Supplemental Table 15. Joint association of CMD status, hPDI, and healthy lifestyle with dementia risk, excluding those with missing data on lifestyle variables.

CMD status	hPDI	Lifestyle score	N	Dementia HR (95% CI)	*p*-value
CMD-free	Low	Healthy	913	0.94 (0.40-2.24)	0.90
		Unhealthy	10,418	0.78 (0.44-1.36)	0.38
	Moderate	Healthy	3,757	0.58 (0.30-1.13)	0.11
		Unhealthy	31,513	0.80 (0.47-1.35)	0.40
	High	Healthy	1,472	Reference	
		Unhealthy	8,846	0.61 (0.35-1.09)	0.09
CMDs	Low	Healthy	107	0.76 (0.40-7.73)	0.45
		Unhealthy	2,021	1.88 (1.02-3.45)	0.04
	Moderate	Healthy	443	2.12 (0.99-4.54)	0.05
		Unhealthy	5,004	1.33 (0.76-2.33)	0.32
	High	Healthy	158	0.45 (0.06-3.41)	0.44
		Unhealthy	1,258	1.11 (0.56-2.22)	0.77

A healthy lifestyle includes high physical activity + never smoking + low-to-moderate alcohol consumption. Models were adjusted for age, sex, race, education, energy intake, socioeconomic status, body mass index, hypertension, and *APOE*4 status.

Abbreviations: CI = confidence interval; CMD = cardiometabolic disease; hPDI = healthful plant-based diet index; HR = hazard ratio.
